# Protein fusion tags for efficient expression and purification of recombinant proteins in the periplasmic space of *E. coli*

**DOI:** 10.1007/s13205-016-0397-7

**Published:** 2016-02-04

**Authors:** Ajamaluddin Malik

**Affiliations:** Department of Biochemistry, Protein Research Chair, College of Science, King Saud University, PO Box 2455, Riyadh, 11451 Saudi Arabia

**Keywords:** Fusion protein, Periplasmic space, Protein folding, Solubility enhancer

## Abstract

Disulfide bonds occurred in majority of secreted protein. Formation of correct disulfide bonds are must for achieving native conformation, solubility and activity. Production of recombinant proteins containing disulfide bond for therapeutic, diagnostic and various other purposes is a challenging task of research. Production of such proteins in the reducing cytosolic compartment of *E. coli* usually ends up in inclusion bodies formation. Refolding of inclusion bodies can be difficult, time and labor consuming and uneconomical. Translocation of these proteins into the oxidative periplasmic compartment provides correct environment to undergo proper disulfide bonds formation and thus achieving native conformation. However, not all proteins can be efficiently translocated to the periplasm with the help of bacterial signal peptides. Therefore, fusion to a small well-folded and stable periplasmic protein is more promising for periplasmic production of disulfide bonded proteins. In the past decades, several full-length proteins or domains were used for enhancing translocation and solubility. Here, protein fusion tags that significantly increase the yields of target proteins in the periplasmic space are reviewed.

## Introduction

Since the advent of production of recombinant proteins, application of therapeutic and diagnostic proteins as biopharmaceuticals was changed remarkably (Walsh 2014). These proteins are required in huge amount and usually can not be obtained from natural sources due to extremely low availability. Moreover, Genetically engineered proteins with special benefits (e.g. Insulin analogs) are as such molecules which can therefore only be obtained via recombinant technology (Walsh 2000, 2006; Sanchez and Demain 2012). *Escherichia coli* was the first and still popularly used host for the fast and economical production of recombinant proteins (Vincentelli and Romier 2013; Chance et al. 1981; Choi and Lee 2004; Rosano and Ceccarelli 2014; Lebendiker and Danieli 2014). In-depth knowledge of genetic and biochemical pathways of *E. coli* and availability of variety of vectors made is an attractive host for such purposes. Although significant improvements have been made at transcription, translation and translocation, still obtaining soluble and bioactive proteins is a major challenge (Pines and Inouye 1999; Baneyx 1999; Rosano and Ceccarelli 2014).

Secreted proteins such as antibodies, enzymes, hormones etc. are used for therapeutic and diagnostic applications. Secreted proteins having two or more cysteines makes disulfide bonds, which is usually vital for structure formation and bioactivity (Creighton 1997b; Creighton et al. 1995; Clarke and Fersht 1993). The cytosol of *E. coli* is reducing which gives inclusion bodies when such proteins are expressed in the cytosol (Freedman 1989; Hwang et al. 1992; Aslund et al. 1994; Carmel-Harel and Storz 2000; Russel 1995; Messens and Collet 2006). Usually in vitro oxidative refolding is difficult, laborious, time consuming and may be uneconomical depending upon refolding yield (Lilie et al. 1998; Lange and Rudolph 2009; Yamaguchi et al. 2013; Basu et al. 2011). Translocation of these proteins into the *E. coli* periplasm provides favorable environment for oxidative folding due to the presence of disulfide bond folding and isomerization machinery (Gopal and Kumar 2013; Yoon et al. 2010; Choi and Lee 2004). Moreover, proteases are less abundant in periplasm and also its relatively less crowded than cytosol which reduces the chances of proteolysis and ease in the purification of recombinant proteins (Makrides 1996). To secrete proteins into periplasmic space, a translocation signal sequence must be fused at the N terminus of proteins, but only the fusion of signal sequence is not enough for efficient protein translocation (Fekkes and Driessen 1999; Muller et al. 2001). The sequences on mature protein next to the signal peptidase cut site and other parts of mature protein play an important role in the secretion (Lee et al. 1989; Malik et al. 2006). Under such condition, fusion to a full-length periplasmic protein that is well stable, soluble and properly folded is more promising (Table 1).

**Table 1:** Properties of periplasmic fusion proteins

Fusion protein	MW (kDa)	calc. pI	S–S bond	Subcellular location
Ecotin	16	5.94	1	Periplasm
Maltose-binding protein	40.7	5.07	0	Periplasm
Z-domain of protein A	6.6	5.16	0	Secreted
ABD-domain of protein G	6	4.46	0	Secreted
CBD from exonuclease	11.1	8.44	1	Secreted
CBD from endonuclease	10.9	6.07	1	Secreted
Disulfide bond oxidoreductase	21.1	5.42	1	Periplasm
Barnase	12.3	8.88	0	Secreted

Size, calculated isoelectric point, number of disulfide bond and native localization were evaluated

Over two decades of extensive in vivo and in vitro research on protein fusions constructs concluded that fusion tags usually increases the yield and solubility of their fusion partners (Costa et al. 2014; Waugh 2005). Despite all these advancement, still it is difficult to choose the best fusion system for a given protein of interest. In general, selection of fusion tag depends upon the properties of protein of interest itself such as size, stability, and hydrophobicity; the expression site; and the usage of the recombinant protein. After coupling with second protein (fusion tag) the increase in yield and solubility the target proteins varies in each fusions. The detailed mechanism by which fusion proteins improve solubility and yield is not well understood. There is two hypotheses: (a) fusion of a stable or conserved structure to an insoluble recombinant protein may serve to stabilize and promote proper folding of the recombinant protein (Butt et al. 2005) and (b) fusion tags may act as a nucleus of folding “molten globule hypothesis” (Creighton 1997a).

Ideally, an effective periplasmic fusion system should have the following features: (a) efficient translocator; (b) enhance folding and solubility; (c) help in purification; (d) facilitate quantification; (e) minimize proteolysis; (f) no adverse effect on the structure and bioactivity; (g) easy and specific removal of the fusion tag; (h) useful for different classes of proteins and peptides. However, none of the fusion tag is optimal with respect to all of these parameters. Successful examples of each periplasmic fusion proteins are listed in Table 2. In the following sections, merits and demerits of available periplasmic fusion proteins are discussed.

**Table 2:** Examples of protein fusion tag assisted production of recombinant proteins in the periplasmic space of *E. coli*

Fusion protein	Model protein	References
Ecotin	Pepsinogen	Malik et al. (2006)
Ecotin	Proinsulin	Malik et al. (2007)
Ecotin	Octapeptide	Paal et al. (2009)
MBP	Merozoite surface protein I	Planson et al. (2003)
MBP	Nanobodies (singe domain antibody)	Salema and Fernandez (2013)
MBP	Membrane protein U24	Tait and Straus (2011)
MBP	Single chain antibody	Hayhurst (2000)
MBP	Pokeweed antiviral protein	Honjo and Watanabe (1999)
SpA	Insulin like growth factor-II	Hammarberg et al. (1989)
SpA	Alkaline phosphatase	Engel et al. (1992)
SpG	Insulin like growth factor-II	Hammarberg et al. (1989)
SpG	Octapeptide	Stahl et al. (1989)
CBD	Polypeptide	Hasenwinkle et al. (1997)
CBD	Lipase	Hwang et al. (2004)
CBD	Beta-glucosidase	Ong et al. (1991)
CBD	Alkaline phosphatase	Greenwood et al. (1989)
DsbA	Enterokinase catalytic subunit	Collinsracie et al. (1995)
DsbA	Proinsulin	Winter et al. (2000)
Barnase	Cystein knot peptide	Schmoldt et al. (2005)

### Ecotin

Ecotin (*E. coli* trypsin inhibitor) is a homodimeric protein which is naturally localized in the periplasmic space (Table 1). The properties of ecotin make it a promising periplasmic fusion tag. It is moderately small in size (16 kDa monomer), extremely stable (tolerates pH 1.0 and 100 °C for 30 min) and contains one disulfide bond in each subunit (Chung et al. 1983). Due to the presence of disulfide bonds, ecotin undergoes a pathway of oxidative folding.

**Fig. 1:**
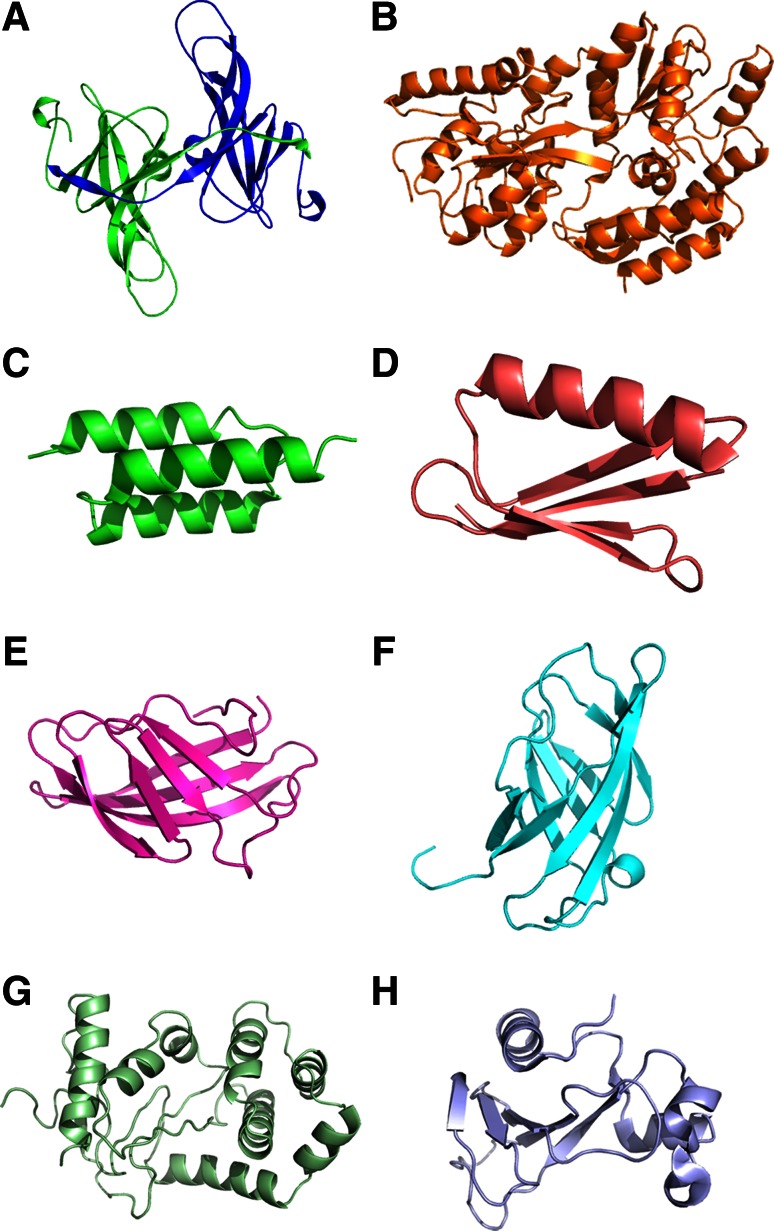
Three-dimensional structure of periplasmic fusion proteins. **a** ecotin (1ECZ), **b** Maltose binding protein (1DMB), **c** Z-domain of protein A (1LP1), **d** ABD-domain of protein G (1EM7), **e** CBD of endoglucanase (1EXG), **f** CBD of exoglucanase (modelled 3D structure), **g** disulfide bond oxidoreductase (1A2J), **h** Barnase (1RNB)

Naturally, ecotin is constitutively expressed (Chung et al. 1983) for the defense of *E. coli* against trypsin like serine proteases in the digestive tract and neutrophil elastase like serine proteases in the blood. Ecotin had no metabolic role or interaction with other proteins in *E. coli* (Eggers et al. 2004). The C termini of each monomer in dimeric ecotin protrude in opposite directions (Fig. 1a), which will allow folding of passenger proteins at each end without steric hindrance. Strong affinity of ecotin’s for trypsin like serine protease will facilitate ecotin fusion protein to purify via affinity chromatography. Ecotin’s binding surface has been already randomized (Stoop and Craik 2003) to reduce its affinity to zymogens of serine proteases, which would help to elute ecotin fusion proteins under softer conditions.

Moreover, model protein in the ecotin fusion system can be quantatively measured in a very sensitive trypsin inhibition assays (Kang et al. 2005). Even in the cytosol ecotin is stable and active; which makes it suitable candidate to be used as cytoplasmic fusion tag (Kang et al. 2005). Ecotin can also be produced in monomeric native state after removal of the last 10 residues (Pal et al. 1996) Thus, ecotin fusion protein in monomeric state is feasible. Ecotin fusion tag have been used for efficient translocation, solubility enhancement and purification of proteins and peptides (Paal et al. 2009; Malik et al. 2006, 2007).

### Maltose-binding protein

Maltose-binding protein (MBP) is cysteine-less relatively large (40.6 kDa) periplasmic protein (Fig. 1b) (Duplay et al. 1984). It is known for its noteworthy solubility enhancement when it is fused at the N terminus of model proteins (Raran-Kurussi et al. 2015; Raran-Kurussi and Waugh 2012; Sachdev and Chirgwin 1998). MBP has been frequently utilized for cytosolic expression but due to its natural periplasmic localization, it is also utilized as periplasmic fusion tag for enhancing secretion, solubility as well as purification of target proteins (Salema and Fernandez 2013; Planson et al. 2003). In certain cases, it was found that MBP attains natively folded state and remains soluble while the passenger proteins could not attained properly folded state and exist as in the state of soluble aggregates (Nallamsetty et al. 2005; Nomine et al. 2001; Sachdev and Chirgwin 1999). The affinity of MBP for maltose is ~1 μM which allowed to purify MBP fusion protein through affinity chromatography (Betton and Hofnung 1996). Moreover, MBP is thermodynamically moderately stable with the* T*_m_ of 62.8 °C at pH 8.3 (Novokhatny and Ingham 1997) and individual components of MBP fusions are slightly more stable than their counterparts in the fusion protein (Blondel et al. 1996).

### Staphylococcal protein A

Staphylococcal protein A (SpA) is a surface protein of Gram-positive bacterium *Staphylococcus aureus* which has strong affinity and high specificity for constant (Fc) part of human immunoglobulins as well as large number of other animals (Eliasson et al. 1988; Cedergren et al. 1993). SpA is a highly soluble 31 kDa protein. Chemically denatured SpA renatures efficiently which assists refolding of the target protein in the SpA fusion system (Samuelsson et al. 1991). SpA is a cysteine-less protein, thus abolishing the chances of interference in disulfide bond formation with fused protein of interest (Kashimura et al. 2013; Uhlen et al. 1984). The gene of SpA is highly repetitive which consists of signal sequence followed by five small highly similar domains (E, D, A, B and C) and C terminal membrane anchoring sequence. The B-domain has been engineered to create smaller variants (7 kDa) of SpA, called as Z-domain (Nilsson et al. 1987). Depending upon localization requirements of the target protein, large number of expression plasmids with or without signal sequences for the production of single Z-domain (7 kDa) or double Z-domains (14 kDa) fusions (Fig. 1c) has been developed (Nilsson et al. 1994, 1996; Hammarberg et al. 1989; Stahl et al. 1989). The fusion protein with Z-domain was more efficiently translocated in comparison to full length SpA proteins (Nilsson et al. 1997).

### Streptococcal protein G

Streptococcal protein G (SpG) present on the *streptococci* surface is a bifunctional receptor and capable of binding with both IgG and serum albumin from different species with different affinities (Nygren et al. 1988). The IgG and albumin binding regions are structurally separated on the SpG. The serum albumin binding region is known as ABD (albumin-binding domain), consists of three binding motifs (each ~5 kDa) (Fig. 1d). Depending upon the localization of the target proteins, ABD with or without signal sequence has been used for expression of fusion protein. Subsequently, fusion proteins were purified via HSA-affinity chromatography in one-step (Hammarberg et al. 1989; Larsson et al. 1996; Stahl et al. 1989).

### Cellulose binding domain (CBD)

Nearly 111 residues from endoglucanase (Fig. 1e) and 100 residues from exoglucanase (Fig. 1f) of *Cellulomonas fimi*, which has high affinity for cellulose, have been used for translocation to periplasmic space and solubility enhancement of target proteins (Gilkes et al. 1988, 1992; Warren et al. 1986; Hwang et al. 2004; Hasenwinkle et al. 1997; Creagh et al. 1996; Ong et al. 1991). The purification of cellulose binding domain fusion protein was achieved via relatively inexpensive ligand matrix (cellulose) (Greenwood et al. 1989, 1992; Ong et al. 1991).

### Disulfide bond oxidoreductase

Disulfide bond oxidoreductase (DsbA) is the key enzyme of periplasmic oxidoreductive system (Fig. 1g). It facilitates correct disulfide bond formation via intra- and intermolecular catalysis (Bardwell et al. 1993). In biotechnological applications, target proteins having multiple disulfide bonds (enterokinase catalytic subunit, proinsulin) were fused at the C terminus of DsbA to enhance disulfide bond formation as well as stabilize unfolded target protein via its polypeptide binding site (Collinsracie et al. 1995; Winter et al. 2000). After fusion with DsbA, these proteins were obtained in the well-folded soluble state in the periplasmic space. DsbA is a potent protein thiol oxidase. It has been observed in vitro experiments that DsbA causes non-native disulfide bond formation in proteins having multiple disulfide bonds (Hirudin, BPTI) (Wunderlich and Glockshuber 1993; Zapun and Creighton 1994). Also, in vivo co-expression of DsbA resulted in inclusion bodies formation of IGF-I (Joly et al. 1998).

### Barnase

Barnase is an enzymatically inactive variant (H102A) of extracellular RNAse from *Bacillus amyloliquefaciens* (Fig. 1h). It is monomeric, cysteine-less protein of relatively small size. For biotechnological applications, enzymatically inactive variant of RNAse was used as a fusion protein to enhance the secretion of cysteine-knot peptides in the periplasmic space. It was found that majority of the cysteine-knot peptides were in the native state when fused with barnase (Schmoldt et al. 2005). Moreover, the Barnase fusion protein could be purified via immobilized barstar (Barnase inhibitor) in single step (Schmoldt et al. 2005).

## Conclusion

Every protein is unique and due to their different applications such as academic research, diagnostic or therapeutic usage, the quantity and purity level vary. Therefore, no single fusion tag will address every requirement. Fusion tags are helpful in enhancing their solubility and stability. Protein fusion tag with μM-nM ligand affinity generally results in 90–99 % purity after affinity chromatography. Removal of protein fusion tag and producing recombinant protein with authentic N terminal adds another layer of complexity. When considering which protein fusion to use, important queries should keep in mind such as: nature of protein itself, how much protein required, application of protein, is fusion tag removal necessary or not, how much additional residues could be tolerated at N terminal? To remove most part of the fusion protein, highly specific protease cleavage site (TEV protease, thrombin, enterokinase, etc.) could be placed in the linker region between fusion tag and model protein. Also, non-specific proteases such as trypsin could be used to generate authentic N terminus as demonstrated in the case of Ecotin-proinsulin fusion protein (Malik et al. 2007). If authentic N terminus is must for the application, ubiquitin fusion technology could be used as successfully demonstrated in ecotin–ubiquitin–peptide fusion system (Paal et al. 2009).
